# Modern Clinical Trials: Seamless Designs and Master Protocols

**DOI:** 10.1002/cam4.71858

**Published:** 2026-04-23

**Authors:** Abigail Burdon, Thomas Jaki, Xijin Chen, Pavel Mozgunov, Haiyan Zheng, Richard Baird

**Affiliations:** ^1^ MRC Biostatistics Unit University of Cambridge Cambridge UK; ^2^ University of Regensburg Regensburg Germany; ^3^ Department of Mathematical Sciences University of Bath Bath UK; ^4^ Department of Oncology University of Cambridge Cambridge UK

**Keywords:** adaptive designs, efficient trials, interim analysis, master protocols, seamless phase II/III

## Abstract

**Background:**

Drug development is often inefficient, costly and lengthy, yet it is essential for evaluating the safety and efficacy of new interventions. Compared with other disease areas, this is particularly true for Phase II/III cancer clinical trials where many drug candidates fail to advance and reduced regulatory approvals are being seen. In response to these challenges, seamless clinical trials and master protocols have emerged to streamline the drug development process.

**Methods:**

Seamless clinical trials, characterised by their ability to transition seamlessly from one phase to another, can lead to accelerating the development of promising therapies while Master protocols provide a framework for investigating multiple treatment options and patient subgroups within a single trial.

**Results:**

We discuss the advantages of these methods through real trial examples and the principles that lead to their success while also acknowledging the associated regulatory considerations and challenges.

**Conclusion:**

Seamless designs and master protocols have the potential to improve confirmatory clinical trials. In the disease area of cancer, this ultimately means that patients can receive life‐saving treatments sooner.

## 1 Background

Development of novel medicines to treat disease is a long and costly process that carries a high risk of failure, with most drug candidates never achieving regulatory approval. This white paper focuses on the area of oncology, where many drug candidates fail to progress through development [1]. Overall success rates are roughly 14% across all disease areas compared with 1% in oncology trials alone [1]. In recent years, an increased emphasis has been placed on data‐informed decision‐making during the development of oncology treatments; in the meanwhile, a more quantitative assessment of developmental risk is used when making decisions about subsequent investments in studies [2].

With a seamless clinical trial design, different stages of development (e.g., Phase I/II, or Phase II/III) are combined in a single trial protocol. This can streamline the study not only operationally but inferentially (i.e., to include data from Phase II in the decision at the end of Phase III) [3]. It can also potentially speed up the development, whereas allowing decisions about particular treatment(s) as promising or not promising ones to be made quickly. Some drug candidates in rare disease areas, such as cancer, may qualify for accelerated approval in which decisions are based on surrogate endpoints such as biomarkers. This kind of regulatory approval may allow for early approval and similarly speed up the drug development process.

Another class of clinical trial design which can improve the efficiency of clinical drug development is master protocols [4], in which multiple treatments and cancer subtypes can be tested within a single trial protocol.

In this paper we discuss different modern approaches that have the potential to make decisions about a treatment's value more efficiently. As a consequence, this might help deliver life‐saving oncology treatments to patients more quickly. Specifically, this work is a focused methodological overview and critical discussion of seamless designs and master protocols.

## 2 Phase II/III Seamless Trials

### 2.1 Seamless Designs in Oncology Settings

In clinical drug development, the initial testing of new drug candidates in Phase II trials helps identify which treatments are looking most promising—and potentially worth testing in (expensive, long) confirmatory Phase III trials.

Conventionally, the learning and confirmatory phases are separate in both their planning and data analyses. One way to accelerate the development process is to combine these phases into a single protocol, so the between‐phase ‘white space’ can be minimised or even removed. Such ‘seamless’ designs require careful planning in advance including decision rules and patient recruitment strategies, etc. Nonetheless, seamless designs are recognised as more efficient in managing development‐related uncertainty. For instance, interim decision gates can be incorporated to terminate the evaluation early if the treatment is either not sufficiently effective or associated with unacceptable side effects.

**FIGURE 1:**
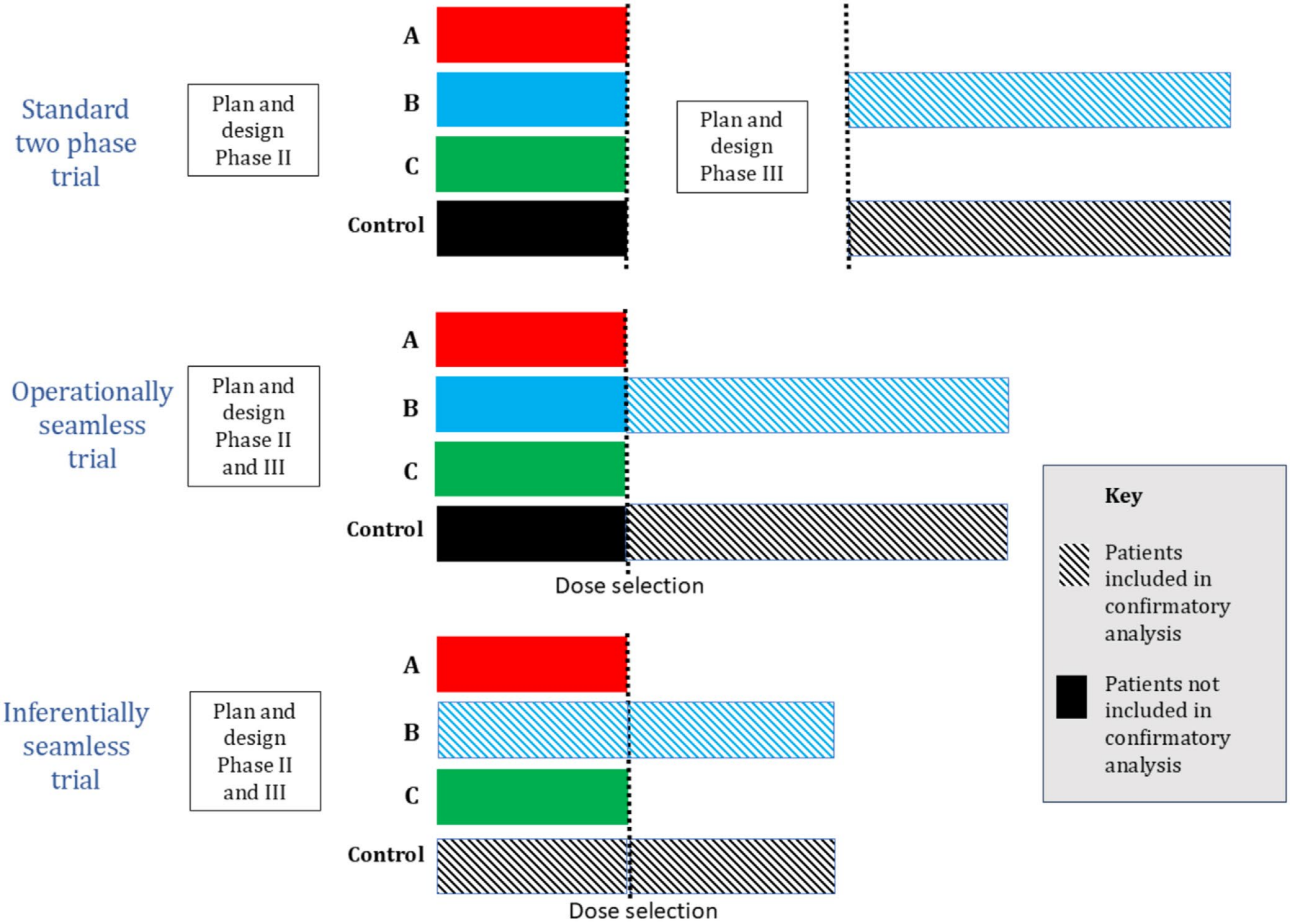
Illustration of inferentially seamless and operationally seamless Phase II/III trials. Length of bars corresponds to the number of patients, which is different per design.

The past decades have witnessed a steady increase in the number of seamless trials. Some prominent examples include the AGILE trial [5] treating patients with glioblastoma, and the Horizon III trial [6] in the disease area of advanced colorectal cancer. Among seamless designs, there are two distinct types: *operationally seamless* and *inferentially seamless*. In an operationally seamless trial, the data collected from the learning and confirmatory stages are kept separate. Here, benefits mainly arise from the combination of the two phases into a single protocol and the reduction in time for decision making. Inferentially seamless trials allow for learning phase data to contribute to the analysis at the confirmatory stage and help establish efficacy. These trials achieve greater efficiency by reusing data from the earlier phase in the confirmatory analysis, thereby reducing the additional sample size needed in the later phase and allowing a given sample size to be reached more readily. Inferentially seamless trials are considered to be a subset of operationally seamless trials (Figure 1).

Seamless designs can facilitate the investigation of a few clinically important questions in parallel within a single study. In Phase II of a clinical development program, investigators may be interested in comparing multiple dose levels or schedules, with the aim being to identify the most efficacious one(s). It is then of interest to compare the experimental treatment, at a chosen dose, with the current standard of care option in a confirmatory Phase III study [7]. Alternatively, Phase II may have been designed with the focus being subgroup identification [8]. In such trials, pre‐defined subgroups of patients are given both experimental and control treatments and the trial would aim to identify which subgroup(s) respond well to treatment. For example, in metastatic breast cancer studies, it is well recognised that treatments specifically targeting HER2 hormone receptors are particularly effective [9]. A seamless Phase II/III study, instead, allows for two questions to be answered succinctly and efficiently without pausing to assess the results of Phase II on completion. In an inferentially seamless trial, the data obtained on patients who received the selected dose or were in the benefitting subgroup(s) can be used in the analysis before and after the adaptation. Seamless trials also allow for added efficiency by exploiting information from short‐term endpoints in Phase II to aid the inference for, and at, the confirmatory phase.

Consequently, the inferentially seamless trial must be set up in a way to respond quickly to interim decisions. For example, the Phase III recruitment strategy should be planned prior to Phase II for all possible combinations of treatment and subgroups, and this is unlikely to be identical across different subgroups. Further, a well‐designed clinical trial builds in flexibility to respond to changes. This can be incorporated by critically evaluating and anticipating possible complexities by computer simulations performed at the outset. The likelihood of unplanned operational changes is even greater for seamless studies. Thus, the need for increased upfront investment and planning is apparent.

In inferentially seamless trials, special attention is required to control the overall Type I error rate because data from different stages are combined to make a single confirmatory conclusion. The null hypothesis typically states that the experimental treatment is equivalent to control in terms of efficacy. When data are analysed at multiple stages (e.g., after both Phase II and Phase III portions), repeated hypothesis testing can increase the chance of a false‐positive finding if conventional significance thresholds are used. To prevent this inflation of Type I error, *alpha‐splitting* methods are applied. This is where the total allowable significance level is divided between stages of the trial, for example by assigning a larger portion to the Phase II and a smaller portion to the Phase III. This ensures that the combined probability of incorrectly rejecting the null hypothesis remains within acceptable limits. Various statistical boundaries can be used to guide this allocation, including Pocock [10], O'Brien and Fleming [11] and Lan and DeMets [12] approaches, which allow differing degrees of conservatism at each interim look. Although alpha‐splitting may result in a modest reduction in statistical power compared with conducting independent Phase II and Phase III trials for the same sample size, it provides efficiency gains by enabling early decisions on futility or efficacy while maintaining strict error control. This trade‐off illustrates a key distinction between seamless and conventional trial designs: seamless approaches optimise resource use and timelines, but require greater upfront planning and statistical rigour to ensure valid inference across stages.

During the analysis of the data from each phase, recommendations can be made to the sponsor regarding how the trial should proceed in the form of go/no‐go decisions. However, to construct these rules, it is necessary for at least one person to have access to data with labelled treatment groups. This unblinding can lead to biased results and compromise the integrity of the drug development. The role of an independent data monitoring committee (IDMC) [13] is therefore imperative to ensure that seamless trials are rigorously conducted. The IDMC may review unblinded data to assess the go/no‐go criteria. Another crucial role of the IDMC is to monitor safety which may require unblinded data at the patient level. For traditional non‐seamless trials, there is no consequence for unblinding data at the end of Phase II since the analysis refreshes and Type I error rates are effectively re‐set. Hence, the compromise of unblinding individuals should be taken into account at the design phase of a seamless clinical trial.

Standard practice in oncology clinical trials is to focus on some long‐term endpoint, such as overall survival (OS) or progression free survival (PFS), as the primary outcome of interest at the end of Phase III. When collecting observations on these long‐term endpoints, the timing of the analyses must be carefully considered. One must ensure that sufficient information is available, whereas being ethical about the length of time that patients are receiving a potentially inefficacious treatment. In many trials, rapidly available short‐term endpoints may be collected and analysed to conduct treatment and/or subgroup selection at the end of Phase II. Data that contribute to these different endpoints can be combined to create a seamless Phase II/III trial which efficiently informs decisions across phases [14]. Hence, we do not need to restrict seamless designs to trials where the Phase II outcome is the same long‐term endpoint as in Phase III. However, using data from multiple endpoints can result in complex analysis methods, thus requiring increased planning. For example, trialists are warned of introducing bias and inflating Type I error rates when the endpoints are correlated and one solution is to separate the data into non‐overlapping time periods for each endpoint [15]. This work is naturally extended to incorporate Phase IV, time‐to‐event endpoints in master protocol studies [16]. Although the statistical challenges are heightened, there is much to be gained when we allow endpoints to differ across phases of seamless trials.

Seamless designs attract heightened regulatory scrutiny and introduce considerable logistical complexity compared with conventional separate‐phase trials. Regulators require a clear prespecification of adaptation rules, justification of statistical methods for controlling Type I error rates, and simulation‐based evaluation of operating characteristics to ensure the validity of inferences [17, 18]. These designs place significant operational demands on sponsors, investigators, and data monitoring committees, who must coordinate continuous recruitment, adaptive decision‐making, and data integrity across phases without interrupting trial conduct. Despite these challenges, these designs hold strong potential to shorten development timelines and reduce patient exposure to ineffective treatments.

### 2.2 Example: Horizon III

The Horizon III trial [6] was an inferentially seamless Phase II/III study which compared a new treatment, cediranib with mFOLFOX6, against the standard of care, bevacizumab with mFOLFOX6, in patients with advanced colorectal cancer. During Phase II, patients were randomised among three arms to receive 20 mg of cediranib, 30 mg of cediranib or a placebo alternative. At the end of Phase II analysis, the primary outcome, overall response rate (ORR) was reported and the IDMC concluded that the treatment arm of 20 mg of cediranib met predefined safety and efficacy criteria. At the start of Phase III, patients who were previously enrolled in Phase II on either 20 mg of cediranib or placebo continued treatment and newly enrolled patients were randomised to either of these two treatment arms. Furthermore, patients who were previously receiving 30 mg of cediranib were unblinded and given the opportunity to be re‐randomised into the trial. At the end of Phase III analysis, the primary outcome was PFS, which did not meet the predefined inferiority limit. Hence, it was concluded that cediranib activity was comparable to that of bevacizumab. Importantly, there were 225 patients whose data contributed towards decisions in both Phases II and III and it was estimated that the drug was made available roughly 1–2 years sooner [6] than a conventional trial which does not incorporate the seamless aspect.

In summary, seamless designs primarily focus on improving the efficiency of sequential drug development by integrating exploratory and confirmatory phases within a single protocol. In contrast, master protocols evaluate multiple hypotheses, treatments, or patient populations under a unified trial infrastructure. Moreover, within each research question or sub‐study, elements of seamless design can still be incorporated to enable efficient transitions between stages. The following section therefore builds on the principles discussed for seamless designs and examines how these ideas are applied within platform, basket and umbrella trials, collectively known as master protocols.

## 3 Master Protocols

### 3.1 Different Types of Master Protocols

The most frequently used clinical trial design is the *two‐parallel‐group design*, in which participants are randomised to one of two treatment groups and then followed over time for assessment of outcomes. There are situations when the evaluation of multiple experimental treatments in parallel is desired. One approach would be to do a series of two‐parallel‐group trials, each one comparing one experimental treatment to the control. On the other hand, efficiencies can be anticipated by evaluating the multiple treatments in a *multi‐arm design*. Arms refer to treatments that include the investigational drug candidate at one or more doses, and one or more control treatments such as placebo and/or an active comparator. In this design, patients are assigned to one of several experimental treatments or controls and will stay in their assigned treatment arm for the duration of the study.

Taking this one step further, we come to master protocols that allow for multiple research questions to be answered under one unifying trial protocol. The benefits include the assessment of multiple treatments and/or patient subgroups, enabling an ongoing trial to add or drop treatments and subgroups. Generally, these designs go beyond the multi‐arm trial due to their time‐effective features to further expedite the clinical drug development process. Table 1 briefly describes the master protocol designs that we shall discuss in this paper.

**TABLE 1:** Trial design definitions and examples in oncology.

Type of trial design	Definition	Example of a trial that successfully implements this design	Example of a trial that would not work well
Seamless	Combines exploratory and confirmatory stages within a single protocol to reduce development time and allow early decisions based on interim results	AGILE [5] in glioblastoma integrates dose‐finding and confirmatory stages, enabling early graduation of effective regimens	CheckMate 143 [19] in glioblastoma: a conventional two‐phase approach was required due to uncertainty in dosing and mechanism, making a seamless design unsuitable
Platform	Multiple experimental treatments assessed in parallel and further treatments can be added and/or removed after the study has started	I‐SPY2 [20] evaluates multiple agents in breast cancer with shared controls, allowing fast learning for different treatments	Pancreatic cancer trial [21]: too few eligible patients to sustain multiple arms or shared control groups
Basket	Tests the effectiveness of a new treatment in patients of various cancer types, sharing a common mutation or biomarker, to which the new treatment targets	VE‐BASKET [22] assessed vemurafenib in diverse cancers harbouring the BRAF V600 mutation, efficiently testing a molecularly targeted therapy across histologies	Docetaxel [23] lacks a single molecular target, so cross‐tumour pooling would not be biologically meaningful
Umbrella	Patients are enrolled of the same cancer type but different gene mutations. The trial evaluates the effectiveness of multiple treatments that are linked with these mutations and biomarkers	Lung‐MAP [24] stratifies patients with squamous non‐small‐cell lung cancer into sub‐studies by genomic profile, enabling evaluation of multiple targeted therapies	Pancreatic cancer trial [25] has very low prevalence of mutations which can be targeted by treatment so very few patients would be eligible

For each of the master protocol designs, the null and alternative hypotheses are more complicated than both the two‐parallel‐group designs and multi‐arm designs, as there are a number of possibilities to consider [26]. The considerations for these trials require additional attention as the number of treatment arms, subgroups and hypotheses are increased. Efficiency gains can also be made by combining types of master protocols, for example we can have Basket type trials sequentially joining an overarching platform and also Umbrella and Basket methodologies can be combined into a single trial protocol.

### 3.2 Platform Trials

**FIGURE 2:**
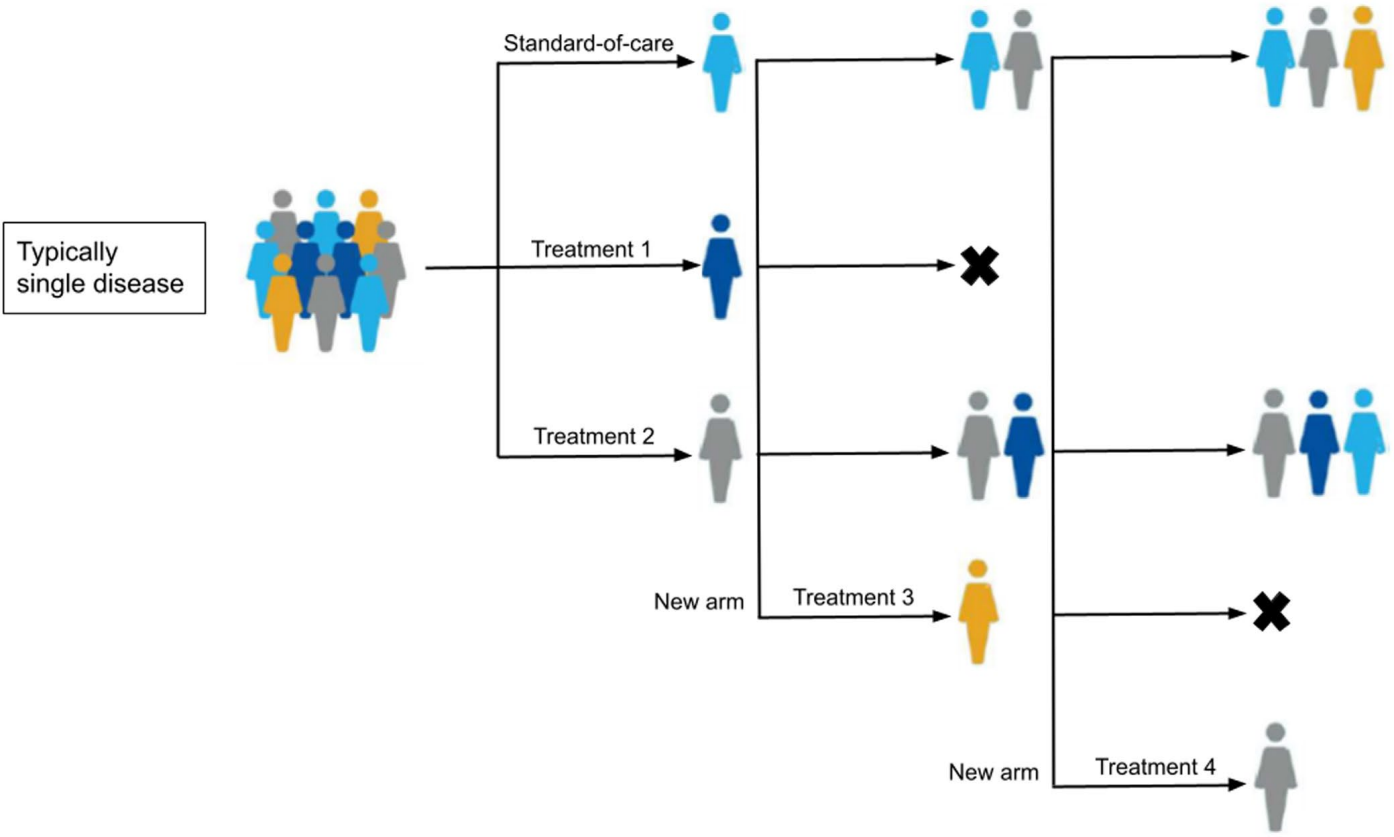
Illustration of platform trials. Interim analyses allow for adaptation: Ineffective arms may be dropped while promising new interventions can be added without stopping the overall trial. Colours indicate different mutations which may or may not determine treatment allocation.

Platform trials are one of the new approaches to clinical research which address the need for enhanced operational efficiency in the modern era of increasingly specific cancer subpopulations and decreased resources to study treatments for individual cancer subtypes in a traditional way [27]. A platform trial comprises a platform in which multiple treatments or treatment combinations are evaluated against a common control for a single disease in homogeneous patient populations in a perpetual manner. The focus of platform trials is on the disease, including several disease subtypes, rather than any particular experimental therapy. Investigated treatments are allowed to enter or leave the platform, often according to pre‐specified decision rules. Figure 2 presents an illustrated example of platform trials and shows how treatments can be added and/or dropped as the trial progresses.

Platform trials have been successfully implemented or are currently being planned in a variety of diseases including breast cancer [28, 29], lung cancer [30, 31, 32] and brain cancer including both Phase II and Phase III settings [27, 33]. There are some prominent platform trials such as the Phase II I‐SPY 2 [34, 35] and the multi‐arm multi‐stage (MAMS), STAMPEDE, used in prostate cancer [36, 37].

Within platform trials, different treatments can be dropped for futility and be added as they become available. Thus, allowing for exploring combinations of treatments and directly comparing competing treatments, both of which are commonly ignored in premarket settings. On the other hand, traditional trial designs are proposed by a single sponsor to evaluate a prespecified and generally limited number of treatments in a homogeneous group of patients. The sharing of resources in platform trials among different sponsors can greatly reduce costs and increase statistical efficiency. This type of trial design has demonstrated great potential for the need for enhanced operational efficiency across the broad spectrum of clinical research. First, not all experimental treatments need to be included in the randomisation scheme at the start of the trial, minimising starting delays. Recruitment speed can be improved compared to starting a new trial from scratch as patients have an increased chance of being allocated an experimental treatment. Platform trials also have the ability to adapt to time trends, for example allowing the standard of care to be updated with accumulating evidence. Finally, smaller sample sizes are required due to a shared control group.

Despite these benefits that have been discussed widely in the literature, corresponding challenges have attracted attention during the implementation of platform trials. One key issue concerns *regulatory acceptance*, as these innovative designs often depart from traditional two‐arm confirmatory frameworks. Regulatory agencies such as the U.S. Food and Drug Administration (FDA) and the European Medicines Agency (EMA) have expressed support for adaptive and platform approaches in principle, but emphasise the need for robust pre‐specified protocols, simulation‐based operating characteristics, and transparent decision rules to ensure interpretability and credibility of results [17, 18]. When new treatment arms are introduced, randomisation ratios adapted, and control arms are shared, this raises concerns about statistical independence. Consequently, early and continuous dialogue with regulators has been strongly recommended for deciding on design features and controlling bias [4, 38].

Another critical consideration is *error rate control*. In conventional two‐arm trials, the probability of a false‐positive result (Type I error rate) is easily maintained at a nominal level. However, in platform trials, multiple experimental arms are evaluated, creating multiple opportunities for false‐positive findings if unadjusted statistical tests are used. Without proper control, the *family‐wise error rate* (FWER) across all comparisons can exceed conventional thresholds. Various statistical strategies have been developed to maintain the overall Type I error [39]. Some examples include multiplicity adjustments, where the *p*‐value threshold is adjusted for each hypothesis, and combination testing, where the stage‐wise test statistics are combined using pre‐specified combination functions.

### 3.3 Basket Trials

**FIGURE 3:**
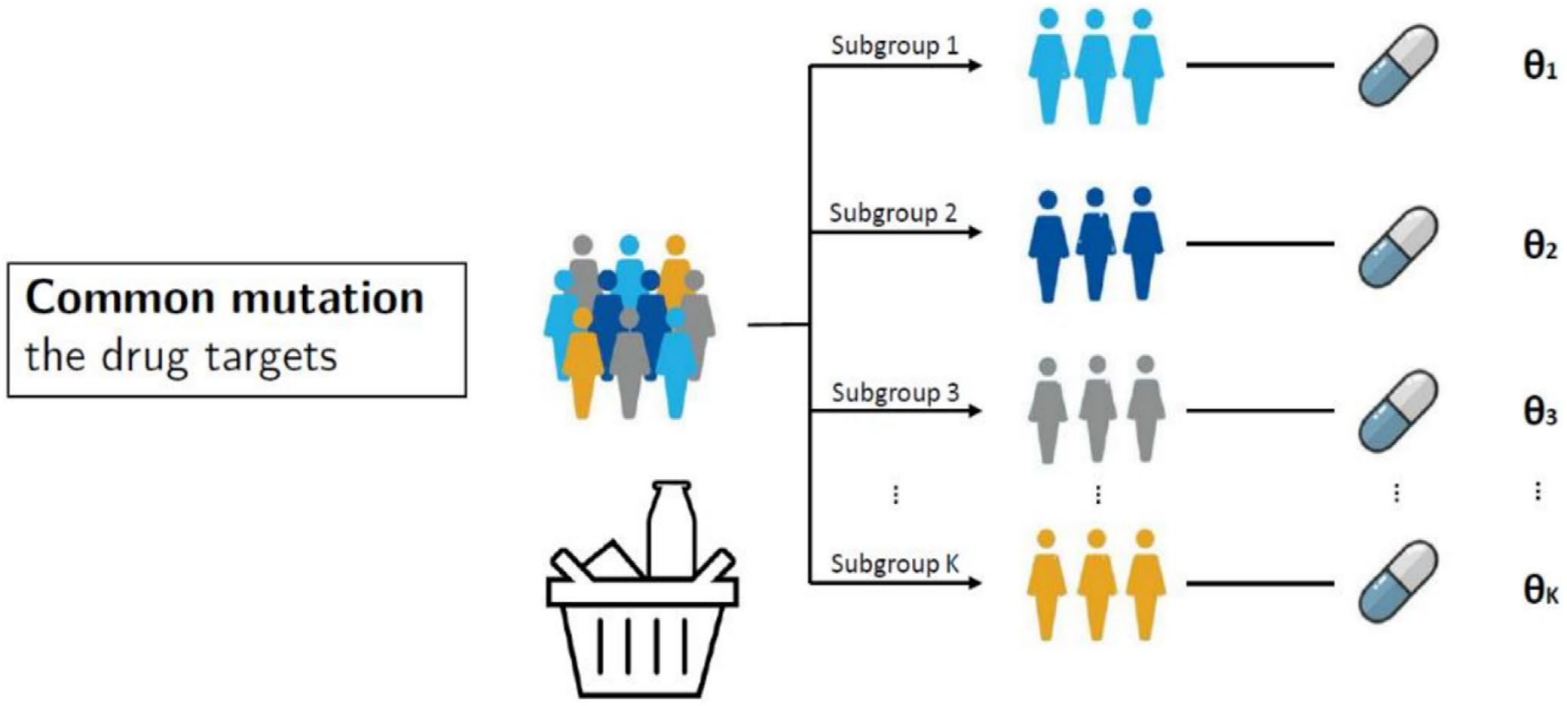
Illustration of basket trials. A single experimental therapy is evaluated across multiple different cancer types that share a common molecular alteration. Rather than grouping patients by tumour site, the trial enrols based on a shared biomarker.

Basket trials [40] involve testing a single new treatment across multiple diseases/cancer types in a single clinical trial protocol. The application in oncology involves enrolling patients with a certain molecular feature (e.g., DNA mutation), irrespective of the location or origin of cancer. Figure 3 gives a graphical illustration of basket trials. One representative example is a Phase II basket trial (NCT01524978) evaluating the efficacy of vemurafenib in patients with BRAF V600‐mutant, a targetable oncogene in various nonmelanoma cancers [22]. This vemurafenib basket trial comprises seven sub‐studies defined by distinct cancer histologies, with the results suggesting preliminary vemurafenib activity in some, but not all, nonmelanoma cancers.

Considering such patient heterogeneity, analysis of basket trials is often directed towards the reporting of the sub‐study specific treatment effects. Although stand‐alone analyses fully acknowledge the heterogeneity, regarding the sub‐studies in isolation means low‐powered tests due to their small sample sizes. Many sophisticated statistical methods have been proposed to enable borrowing of information [40, 41, 42, 43, 44], as those could be justified by the biological basis that all sub‐studies share a commonality (e.g., BRAF V600‐mutant in the vemurafenib trial). Major advantages of such approaches generally include a higher statistical power to detect early efficacy of the treatment, and a smaller sample size [45] than is required by standard‐alone analyses to ensure the same level of decision accuracy.

Basket trials are proven to possess enhanced efficiency in Phase II settings for the simultaneous evaluation of multiple patient subgroups. Further efficiency may be gained by incorporating interim monitoring, as subgroups that are less likely to respond can be dropped at the mid‐course. More complex configurations of basket trials that accommodate multiple targets and/or agent combinations have also been performed (e.g., see Section 3.5 below).

### 3.4 Umbrella Trials

**FIGURE 4:**
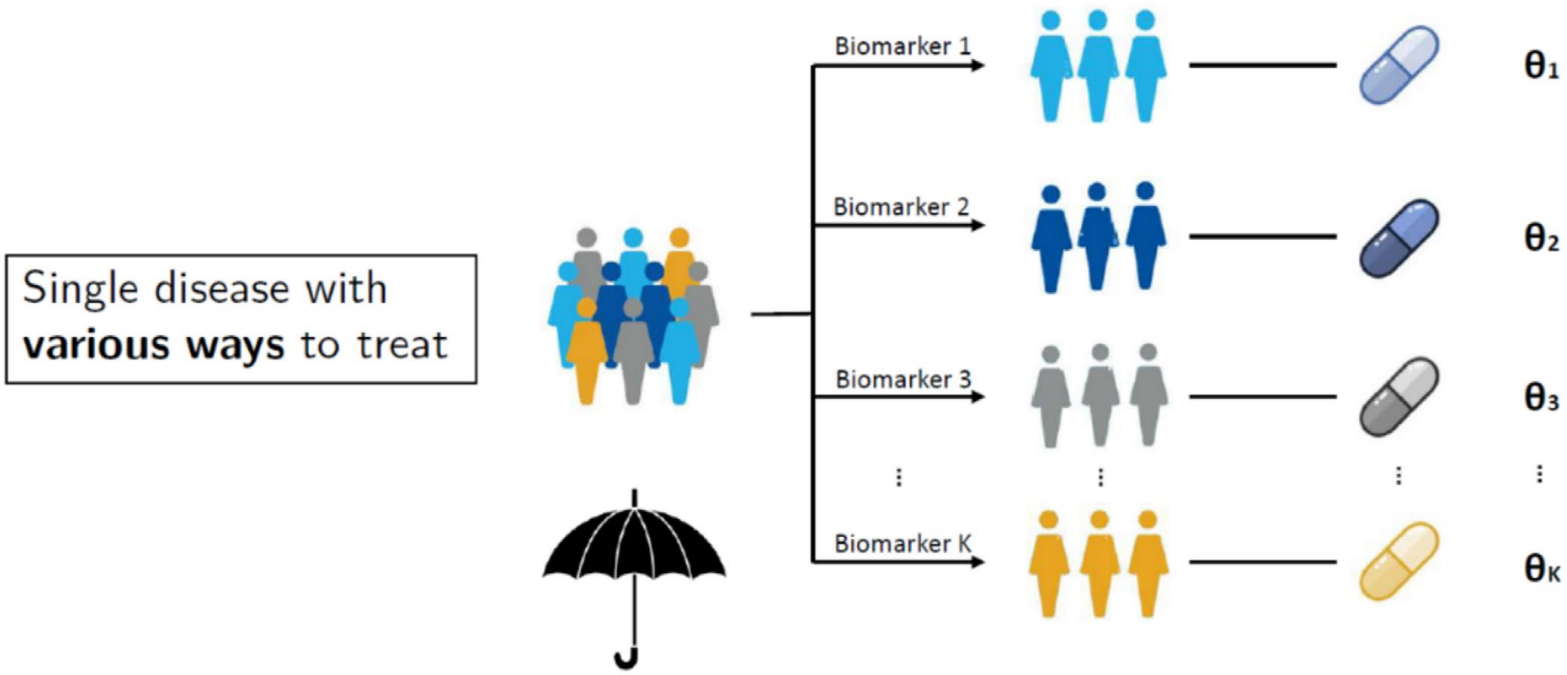
Illustration of umbrella trials. A single disease is stratified into biologically defined subgroups based on predictive biomarkers. Each subgroup is matched to a different targeted therapy, allowing evaluation of multiple precision treatments within one disease type.

Umbrella trials are another major type of precision cancer medicine trials [46], in which patients with a single tissue type of cancer (e.g., breast cancer) have molecular profiling performed on their tumours to test for the presence of multiple different biomarkers. Accordingly, umbrella trials involve a few individualised treatments to see if they might each be matched to the biomarker. Patients with tumours positive for biomarker A are matched to drug candidate A, those positive for biomarker B to drug candidate B, and so on. Figure 4 shows an example illustration of umbrella trials.

The molecular profiling performed in many umbrella trials involves the DNA sequencing of tumours to test for potentially targetable gene mutations which may be driving the growth of those cancers. A pioneering example of such studies is the SAFIR‐01 trial [47], in which 423 patients with metastatic breast cancer consented to have a fresh tumour biopsy for profiling by comparative genomic hybridisation and Sanger gene sequencing. One hundred and ninety‐five of these patients were found to have targetable genomic alterations and 55 were started on molecular targeted therapy matched to their tumour.

Umbrella trials can be highly efficient since they permit the parallel testing of multiple therapies in a single clinical trial protocol. One of the major challenges of umbrella trials is that many targetable gene mutations are uncommon and may be found in only 1%–5% of patient cancers. Therefore, a huge logistical effort is required to screen enough patients to find those with these alterations which can be successfully targeted.

**FIGURE 5:**
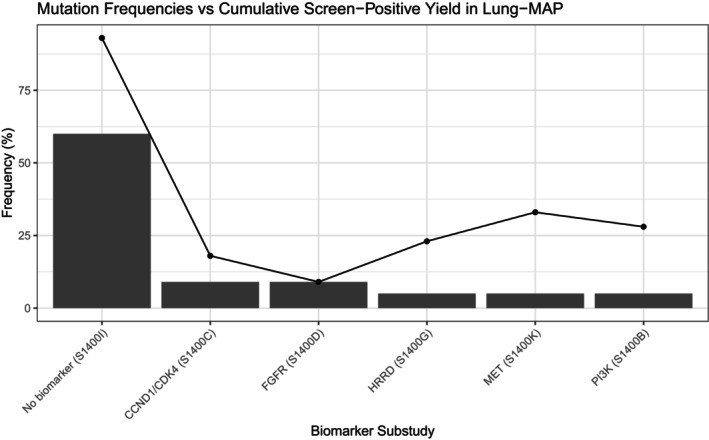
Barchart of mutation frequencies of individual mutations in the Lung‐MAP study [24]. Points show the cumulative frequencies when many mutations are included in the same umbrella trial.

To illustrate the challenge of biomarker prevalence in umbrella trials, we calculated the individual and cumulative frequencies of genomic subtypes from Lung‐MAP (SWOG S1400). Although each biomarker‐defined cohort represented only 2%–6% of screened patients individually, the cumulative frequency across all targeted sub‐studies exceeded 20%. Figure 5 shows the increase in population coverage as additional biomarker‐defined subgroups are included, highlighting how umbrella designs can efficiently study multiple rare alterations within a single screening platform.

### 3.5 Example: I‐SPY 2 Trial

Investigation of serial studies to predict your therapeutic response with imaging and molecular analysis 2 (I‐SPY 2) is a Phase II trial used to screen neoadjuvant treatment of women with locally advanced breast cancer [20]. Multiple therapies are tested as neoadjuvant treatments in I‐SPY 2. Unlike ‘treatment‐focused’ designs, I‐SPY 2 is a ‘disease‐focused’ master protocol trial [48], which is in the context of several sub‐studies for different disease subtypes [49]. Response‐adaptive randomisation is used to assign patients to the most promising treatment in their respective genetic breast‐cancer subgroups while maintaining a sufficient number of patients assigned to the standard of care [4]. For this reason, this biomarker‐based Bayesian adaptive design achieves improved efficiency in identifying improved treatment regimens for patient subsets on the basis of molecular characteristics (biomarker signatures) [20]. In the trial, regimens will be dropped for reasons of futility if they show a low probability of improved efficacy with any biomarker signature. New drug candidates will enter as those that have undergone testing are graduated or dropped.

### 3.6 Example: Basket of Baskets Trial

The Basket of Baskets trial (‘BoB’, NCT) is an example of a Master Protocol in which patients first undergo broad DNA‐sequencing of their cancer (‘BoB iProfiler’). Patients with certain targetable DNA abnormalities can then be offered a therapy matched to their tumour profile. There are multiple different targeted therapies available within the trial protocol—each effectively its' own basket trial—hence ‘Basket of Baskets’. Treatments include an immune checkpoint inhibitor module for patients with tumours bearing genetic features thought to increase the chance of immunotherapy response (including high Tumour Mutational Burden and individual mutations in genes involved in DNA damage repair). New treatment arms are added to the BoB trial protocol as protocol amendments, making efficient use of the existing trial protocol and molecular profiling infrastructure.

## 4 Regulatory Approvals

Full regulatory approval for drug candidates which treat cancer requires solid evidence of safety and therapeutic effectiveness based on adequate and well‐controlled clinical trials. Regular approval was the only pathway supported by the US Food and Drug Administration (FDA) until 1992. However, during the height of the HIV/AIDS crisis, it became clear that patients with life‐threatening diseases could not wait for the lengthy completion of conventional trials. To address this urgent unmet medical need, the FDA introduced the Accelerated Approval (AA) pathway allowing earlier access to promising therapies.

Accelerated approval is an accelerated pathway to regulatory approval for drug candidates that treat serious or life‐threatening conditions including cancer. In order for a drug candidate to qualify for AA, it must show an effect on a surrogate endpoint (such as tumour shrinkage or biomarker response) which is reasonably likely to predict significant clinical benefit—compared to existing available therapies.

Although the AA pathway enables patients to access potentially life‐saving treatments years earlier, it also carries evidentiary risk if the surrogate endpoints fail to correlate with actual patient outcomes. Long‐term FDA analyses have shown that the majority of AA indications do ultimately confirm benefit [50], with only a small fraction withdrawn due to failure to verify clinical efficacy. However, in their review Chen et al. [51] found that only 55% of accelerated approvals based on tumour response rate were converted to full approvals. This highlights the complexity of oncology drug development and reinforces the need for robust evidence generation and well‐defined regulatory decision frameworks.

Similar systems for expedited regulatory review exist in other parts of the world (see Table 2). In Europe, the European Medicines Agency (EMA) conditional marketing authorisation (CMA), which was introduced in 2006, can be based on less comprehensive evidence at the time of initial authorisation. Although both mechanisms aim to expedite access to therapies addressing serious or life‐threatening conditions, the CMA is time‐limited and contingent on additional data submission, whereas AA grants full approval status conditional on confirmatory trials. The two are therefore conceptually related but legally distinct.

**TABLE 2:** Characteristics of US Food and Drug Administration (FDA) and European Medicines Agency (EMA) expedited programs.

Expedited program	Year introduced	Eligibility criteria	Benefits and key features
*FDA*			
Fast track	1987	Drug candidate addresses an unmet medical need for a serious condition	Early and frequent communication with FDA; rolling review of data
Accelerated approval	1992	Drug candidate provides meaningful advantage over existing therapies and demonstrates effect on a *surrogate* or *intermediate* clinical endpoint reasonably likely to predict clinical benefit	Earlier approval based on surrogate endpoint; requires post‐approval confirmatory trials to verify benefit
Priority review	1992	Drug candidate offers significant improvement in safety or effectiveness	Reduced review time (6 months vs. standard 10 months)
Breakthrough therapy	2012	Drug candidate shows substantial improvement on clinically significant endpoint over available therapies	Intensive FDA guidance and organisational commitment to expedite development and review
*EMA*			
Accelerated assessment	2005	Innovative drug candidate of major public health interest, particularly from a therapeutic innovation perspective	Shortened review time (150 days vs. standard 210 days)
Conditional marketing authorisation	2006	Drug candidate addresses unmet medical needs; benefit of immediate availability outweighs problems associated with incomplete data	Time‐limited authorisation (renewable annually); full marketing authorization based on surrogate endpoint

*Note:* Table modified from Ref. [52].

Both the FDA and EMA have evolved their decision‐making frameworks to accommodate innovative and flexible trial designs, especially in settings where conventional randomised trials are infeasible or unethical. The use of surrogate or intermediate endpoints, adaptive trial methodologies, and real‐world evidence is increasingly accepted when the biological rationale is strong and the unmet medical need is compelling. In oncology, the agencies may judge surrogate endpoints appropriate when they are supported by mechanistic understanding, historical validation, or robust correlation with survival outcomes. These regulatory mechanisms reflect a balance between timely patient access and scientific rigour, recognising that accelerated development must still display sufficient evidence of benefit to patients.

## 5 Discussion

Although a vast number of options of innovative designs are being used in practice and getting the attention, there is no ‘one‐size‐fits‐all’ approach when it comes to which trial design (master or seamless or both) should be used. The best‐suited answer depends on the objective and on a particular trial setting in question. For example, basket designs originally were designed to deal with the problem of small sample size in each subgroup and to enhance the efficiency via borrowing on information across subgroups. However, there should be sufficient scientific rationale and preliminary evidence on why ‘borrowing’ of information might be appropriate between different cancer types (e.g., due to a common mutation). Similarly, although platform trials do provide a great potential for the gain in efficiency, they also impose major inferential challenges (e.g., robustness to time trends, use of non‐concurrent controls, etc.). To make the decision on why the platform trial is appropriate, one should weigh these against the option of conducting a ‘conventional’ trial, which might be more robust to these.

Concerning the choice of the seamless design, the decision whether to choose it should match the overall objective of the trial development. For example, if there is a major uncertainty between Phase II and Phase III in the choice of endpoints, populations, doses, durations, etc., it might be beneficial to conduct two separate trials. If different endpoints are used for Phase II and Phase III trials, one should have a solid understanding of the association between these to answer the relevant research question. As for any adaptive design, the evaluation window for the endpoints and recruitment rates would have a major impact on the benefits such seamless options can provide.

Importantly, neither master protocol nor seamless design should be seen as an option to ‘get away’ with no pre‐planned analyses and ‘following the data’. Regardless of the trial design used, the decision‐making criteria (e.g., to progress to Phase III from Phase II, or to terminate a basket, or to stop trial earlier for efficacy) should be pre‐specified before the trial start.

Alongside methodological, regulatory and funding considerations, it is also important to recognise that patient‐level factors play a key role in determining the real‐world impact of these trial designs. Whether patients are able and willing to take part can depend on their mental health, personal and family circumstances, access to care, travel requirements and the level of support they receive. These factors can influence both participation and outcomes and therefore affect how widely trial results can be applied in practice. In particular, equitable access to clinical trials remains challenging and only a relatively small proportion of patients gain access to seamless designs and master protocol trials. Structural, geographic and socio‐demographic barriers may limit participation and contribute to differences in who benefits from these innovative designs [53]. Although such designs have the potential to improve efficiency, this may not be fully realised without deliberate efforts to address these barriers.

An additional and often under‐discussed issue relates to exclusion criteria, particularly the frequent exclusion of patients with psychological and mental health conditions. In many oncology trials, individuals experiencing anxiety and/or depression are excluded by default even though such conditions may arise as a consequence of a cancer diagnosis. This may introduce bias and alter the study population [54]. Greater attention to exclusion criteria could contribute to more clinically relevant trial designs.

In this paper are some limitations to be acknowledged. The work is primarily descriptive and illustrative and is not intended to be a formal systematic review. Therefore, the selection of examples is not exhaustive but has been chosen to highlight key concepts and practical considerations for seamless designs and master protocols. Further, we have discussed methodological and regulatory considerations for oncology trials; however, the implementation of these innovative designs may vary across different therapeutic areas and healthcare systems.

Finally, the consideration on opting to master protocol and/or seamless designs could be driven by the fact whether the study is industrially or academically funded as this majorly affects the ultimate objective. If planning for these, under the academic funding scheme specifically, one needs to ensure the sufficient funding available to reach a definite conclusion on the research question.

## Author Contributions

**Abigail Burdon:** methodology, investigation, writing – original draft, writing – review and editing, conceptualization. **Thomas Jaki:** conceptualization, investigation, methodology, writing – review and editing, writing – original draft. **Xijin Chen:** conceptualization, investigation, methodology, writing – original draft, writing – review and editing. **Pavel Mozgunov:** conceptualization, methodology, investigation, writing – original draft, writing – review and editing. **Haiyan Zheng:** conceptualization, investigation, methodology, writing – original draft, writing – review and editing. **Richard Baird:** conceptualization, investigation, methodology, writing – original draft, writing – review and editing.

## Funding

This project has received funding from the European Union's Horizon 2020 research and innovation programme under grant agreement no. 965397. HZ's and RB's contribution to this manuscript was supported by Cancer Research UK (RCCCDF‐May24/100001). This report is independent research supported by the National Institute for Health and Care Research (NIHR300576). PM and TJ also received funding from the UK Medical Research Council (MC_UU_00040/03). Infrastructure support is acknowledged from the NIHR Cambridge Biomedical Research Centre (BRC‐1215‐20014) and Cambridge Experimental Cancer Medicine Centre.

## Conflicts of Interest

The authors declare no conflicts of interest.

## Data Availability

Data sharing not applicable to this article as no datasets were generated or analysed during the current study.
